# Phase II study of the oxygen saturation curve left shifting agent BW12C in combination with the hypoxia activated drug mitomycin C in advanced colorectal cancer

**DOI:** 10.1054/bjoc.2000.1138

**Published:** 2000-06

**Authors:** D J Propper, N C Levitt, K O'Byrne, J P Braybrooke, D C Talbot, T S Ganesan, C H Thompson, B Rajagopalan, T J Littlewood, R M Dixon, A L Harris

**Affiliations:** ICRF Medical Oncology Unit, Churchill Hospital, Headington, Oxford, OX3 7LJ, UK; Department of Haematology, Oxford-Radcliffe NHS Trust, Oxford, OX3 9DU, UK; Medical Research Council Biochemical and Clinical Magnetic Resonance Unit, Oxford-Radcliffe NHS Trust, Oxford, OX3 9DU, UK

**Keywords:** bioreductive agent potentiation, MRS, ATP

## Abstract

BW12C (5-[2-formyl-3-hydroxypenoxyl] pentanoic acid) stabilizes oxyhaemoglobin, causing a reversible left-shift of the oxygen saturation curve (OSC) and tissue hypoxia. The activity of mitomycin C (MMC) is enhanced by hypoxia. In this phase II study, 17 patients with metastatic colorectal cancer resistant to 5-fluorouracil (5-FU) received BW12C and MMC. BW12C was given as a bolus loading dose of 45 mg kg^−1^over 1 h, followed by a maintenance infusion of 4 mg kg^−1^h^−1^for 5 h. MMC 6 mg m^−2^was administered over 15 min immediately after the BW12C bolus. The 15 evaluable patients had progressive disease after a median of 2 (range 1–4) cycles of chemotherapy. Haemoglobin electrophoresis 3 and 5 h after the BW12C bolus dose showed a fast moving band consistent with the BW12C-oxyhaemoglobin complex, accounting for approximately 50% of total haemoglobin. The predominant toxicities – nausea/vomiting and vein pain – were mild and did not exceed CTC grade 2. Liver^31^P magnetic resonance spectroscopy of patients with hepatic metastases showed no changes consistent with tissue hypoxia. The principle of combining a hypoxically activated drug with an agent that increases tissue hypoxia is clinically feasible, producing an effect equivalent to reducing tumour oxygen delivery by at least 50%. However, BW12C in combination with MMC for 5-FU-resistant colorectal cancer is not an effective regimen. This could be related to drug resistance rather than a failure to enhance cytotoxicity. © 2000 Cancer Research Campaign


*British Journal of Cancer* (2000) **82**(11), 1776–1782
© 2000 Cancer Research Campaign
DOI: 10.1054/ bjoc.2000.1138, available online at http://www.idealibrary.com on IDEAL®

### Phase II study of the oxygen saturation curve left shifting agent BW12C in combination with the hypoxia activated drug mitomycin C in advanced colorectal cancer

**DJ Propper[1], NC Levitt[1], K O'Byrne[1], JP Braybrooke[1], DC Talbot[1], TS Ganesan[1], CH Thompson[3], B Rajagopalan[3], TJ Littlewood[2], RM Dixon[3] and AL Harris[1]**

[1]ICRF Medical Oncology Unit, Churchill Hospital, Headington, Oxford OX3 7LJ, UK; [2]Department of Haematology, Oxford-Radcliffe NHS Trust, Oxford OX3 9DU, UK; [3]Medical Research Council Biochemical and Clinical Magnetic Resonance Unit, Oxford-Radcliffe NHS Trust, Oxford OX3 9DU, UK

**Summary** BW12C (5-[2-formyl-3-hydroxypenoxyl] pentanoic acid) stabilizes oxyhaemoglobin, causing a reversible left-shift of the oxygen saturation curve (OSC) and tissue hypoxia. The activity of mitomycin C (MMC) is enhanced by hypoxia. In this phase II study, 17 patients with metastatic colorectal cancer resistant to 5-fluorouracil (5-FU) received BW12C and MMC. BW12C was given as a bolus loading dose of 45 mg $kg^{-1}$ over 1 h, followed by a maintenance infusion of 4 mg $kg^{-1}$ $h^{-1}$ for 5 h. MMC 6 mg $m^{-2}$ was administered over 15 min immediately after the BW12C bolus. The 15 evaluable patients had progressive disease after a median of 2 (range 1–4) cycles of chemotherapy. Haemoglobin electrophoresis 3 and 5 h after the BW12C bolus dose showed a fast moving band consistent with the BW12C-oxyhaemoglobin complex, accounting for approximately 50% of total haemoglobin. The predominant toxicities – nausea/vomiting and vein pain – were mild and did not exceed CTC grade 2. Liver $^{31}P$ magnetic resonance spectroscopy of patients with hepatic metastases showed no changes consistent with tissue hypoxia. The principle of combining a hypoxically activated drug with an agent that increases tissue hypoxia is clinically feasible, producing an effect equivalent to reducing tumour oxygen delivery by at least 50%. However, BW12C in combination with MMC for 5-FU-resistant colorectal cancer is not an effective regimen. This could be related to drug resistance rather than a failure to enhance cytotoxicity. © 2000 Cancer Research Campaign

**Keywords**: bioreductive agent potentiation; MRS; ATP

BW12C (5-[2-formyl-3-hydroxypenoxyl] pentanoic acid) is an agent which increases the affinity of haemoglobin for oxygen by stabilizing oxyhaemoglobin in a dose-dependent manner (Beddell et al, 1984). This results in a left-shift in the haemoglobin oxygen saturation curve (OSC), thereby reducing oxygen dissociation at low partial oxygen pressures and so reducing tissue oxygen delivery. The effect of BW12C on oxygen dissociation is particularly marked at low partial pressures of oxygen (Beddell et al, 1984). Hence delivery of oxygen to hypoxic tissues will be most effected by BW12C, and this reduction in oxygen delivery will be much greater than is proportional to the difference in oxygen pressures between hypoxic and normoxic tissues. Tumours have areas of significant hypoxia compared to normal tissues. This is thought to contribute to chemo- and radioresistance (Adams et al, 1997).

Mitomycin C (MMC) is a cytotoxic agent which is active in gastrointestinal cancer. Under reducing conditions it forms alkylating metabolites that produce DNA cross-links (Sartorelli et al, 1994). In vitro, the cytotoxic activity of MMC is increased by hypoxia (Sartorelli et al, 1994). Combining such a bioreductively activated agent with BW12C could enable relative targeting of hypoxic tumours. Tumour xenograft data show that BW12C increases the cytotoxic activity of various bioreductively activated drugs (Cole and Robbins, 1989). In addition, BW12C can protect normal tissue from radiation damage, and mimic hypoxically induced necrosis in certain tumours (Adams et al, 1986; Honess et al, 1992). These effects are presumed secondary to effects on tissue oxygenation.

Because of the possibility that BW12C could potentiate the cytotoxicity of MMC, we performed a phase I study of BW12C in combination with MMC in patients with advanced cancer (Philip et al, 1993). Patients received a bolus loading dose of BW12C over 1 h, followed by a maintenance infusion of 4 mg $kg^{-1}$ $h^{-1}$ for 5 h to prolong the duration of the shift in OSC. MMC 10 mg $m^{-2}$ was administered over 15 min immediately after the end of the bolus of BW12C. The dose-limiting toxicities were myocardial ischaemia and reductions in conscious level. Other toxicities were headache, nausea/vomiting, sinus tachycardia and vein pain. These toxicities were dose-dependent. The maximum tolerated dose (MTD) of BW12C was 50 mg $kg^{-1}$ over 1 h.

Pharmacokinetic analysis provided no evidence that BW12C affected plasma MMC levels. We observed changes in the haemoglobin electrophoretic profile consistent with BW12C-oxyhaemoglobin complexing, and a dose-related left-shift of the OSC of above 40% at the top dose level. Phosphorus magnetic resonance spectroscopy ($^{31}P$ MRS) of exercising calf muscle showed that BW12C treatment increased the breakdown of high energy phosphate stores and reduced pH, consistent with tissue hypoxia.

*Received 1 June 1999*
*Revised 16 February 2000*
*Accepted 17 February 2000*

*Correspondence to:* AL Harris

The aim of this phase II trial was to determine tumour response rates to BW12C in combination with MMC in patients with advanced colorectal cancer, and to further examine the toxicity of the regimen. Levels of BW12C-oxyhaemoglobin complex were measured as an indirect assessment of tissue oxygen delivery. In addition, patients underwent $^{31}$P MRS of liver metastases, to determine whether BW12C could induce tumour hypoxia.

### PATIENTS AND METHODS

### Eligibility criteria

Eligibility criteria for entry included: histologically or cytologically proven colorectal carcinoma with liver metastases and evidence of disease progression, Eastern Co-operative Oncology Group (ECOG) performance status of 0–2, life expectancy of at least 3 months, presence of measurable or evaluable disease, haemoglobin greater than 10 g dl$^{-1}$, white cell count greater than $3.5 \times 10^9$ L$^{-1}$, platelet count greater than $100 \times 10^9$ L$^{-1}$, normal renal function and adequate hepatic function with bilirubin less than 1.5 times normal and transaminases less than 5 times normal. Patients were excluded if they had symptomatic ischaemic heart disease or cerebrovascular disease, had previously received MMC or any chemotherapy or radiotherapy within 4 weeks (6 weeks in the case of nitrosoureas) of commencing this study. Approval to conduct this study was granted by the Central Oxford Research Ethics Committee and conducted according to the declaration of Helsinki. Each patient provided written informed consent.

### Drug administration

BW12C was initially administered as a single agent (cycle 1a) followed a week later by the combination of BW12C and MMC (cycle 1b). Thereafter BW12C and MMC were given in combination, as in cycle 1b, every 3 weeks. BW12C (Wellcome Research Laboratories, Beckenham, UK) was administered via a controlled intravenous (i.v.) infusion through a peripheral venous cannula. A loading dose of BW12C – 45 mg kg$^{-1}$ – was dissolved in 250 ml of isotonic saline and infused over 1 h, followed by a maintenance infusion of 4 mg kg$^{-1}$ h$^{-1}$ for 5 h in 1 litre of isotonic saline. The loading dose was one dose increment below the MTD (50 mg kg$^{-1}$) of the phase I study (Philip et al, 1993).

Mitomycin C MMC (Kyowa; Martindale Pharmaceuticals Ltd, Romford, UK) was administered by i.v. bolus injection of 6 mg m$^{-2}$ (maximum dose 10 mg m$^{-2}$) over 15 min. The injection was given at the completion of the loading infusion of BW12C to coincide with the peak modification of the oxygen saturation curve (Philip et al, 1993). Anti-emetic medication comprised metoclopramide 10 mg orally (p.o.) 4 times daily, with the addition of dexamethasone 8 mg p.o. twice daily for 1 day followed by 4 mg p.o. for 3 days in the event of nausea.

### Assessment of toxicity

Baseline investigations included full blood count with a differential white cell count, serum biochemistry and carcinoembryonic antigen (CEA), urinalysis, chest radiograph and electrocardiogram. Patients were reviewed by a physician before each cycle and new symptoms, signs and ECOG performance status were documented. At each visit, a full blood count with differential white count and serum biochemistry and CEA were performed. Additional tests were performed as appropriate. National Cancer Institute Common Toxicity Criteria (NCI-CTC) were used to grade toxicity.

### Assessment of tumour response

Evaluable and measurable disease sites were assessed before entering the study by physical examination, plain radiography, computerized tomography and magnetic resonance imaging where appropriate. This was repeated every two cycles or at the time of suspected disease progression.

Standard WHO criteria for objective response assessment were employed. Partial responses were defined as a 50% or greater reduction in the sum of the products of the largest perpendicular diameters of all measurable disease sites. Progressive disease was indicated by a greater than 25% increase in the size of at least one measurable lesion, or the appearance of a new lesion. Stable disease was defined as an increase in the disease measurements of less than 25% or a decrease of less than 50%. Patients with progressive disease were withdrawn from the study.

### Haemoglobin electrophoresis

In our phase I study (Philip et al, 1993), BW12C treatment produced a dose-dependent left-shift in the OSC, and concomitant haemoglobin electrophoresis showed a fast running band consistent with the BW12C-oxyhaemoglobin complex. Studies by Beddell et al correlated the percentage of BW12C complexed haemoglobin with amplitude of left-shift for the whole OSC curve (Beddell et al, 1984). This provides data on effects on haemoglobin oxygen saturation for the physiological range of oxygen partial pressures, and a better estimation of physiological effects of the compound than measurement of oxygen saturation at one particular oxygen pressure. Haemoglobin electrophoresis was therefore performed on samples pre-BW12C treatment and during the maintenance infusion to demonstrate adequate BW12C-oxyhaemoglobin binding, indicative of a biologically relevant left-shift of the oxygen saturation curve.

Five millilitres of venous blood in an EDTA containing tube was obtained immediately before administering BW12C and repeated at 3 and 5 h from the start of the loading infusion. Sampling was performed during cycles 1a and 1b. Haemoglobin electrophoresis was performed using a standard technique (Lehmann and Huntsman, 1966). Briefly, a lysate was obtained by washing the whole blood in isotonic saline and adding carbon tetrachloride. The lysate was then applied on a cellulose acetate strip. After complete separation, strips were stained with Ponceau S (BDH, Dorset, UK) and the excess stain was removed by immersion in 3% acetic acid. Spectrophotometry was used to evaluate the percentage of BW12C-oxyhaemoglobin complex relative to the total haemoglobin.

### Magnetic resonance methods

The $^{31}$P MR spectrum of the liver contains signals from ATP and other nucleotides, inorganic phosphate (Pi), phosphomonoester (PME) and phosphodiester (PDE). Information about the energy metabolism is obtained from the relative intensities of the ATP and Pi signals, together with the pH, which can be measured from the relative frequency of the Pi signal. If BW12C causes sufficient hypoxia in the liver that the cells cannot meet their energy needs,

*© 2000 Cancer Research Campaign* 

the Pi:ATP ratio should increase, and the pH decrease. Information about carbohydrate and lipid metabolism can be obtained from the other signals. The PME signal contains contributions from AMP, phosphorylated sugars, and other intermediates of carbohydrate, lipid and phospholipid metabolism (including phosphoethanolamine and phosphocholine). Tumours contain a prominent PME signal, which has been found to consist largely of phosphoethanolamine (Negendank, 1992), and may signify differences in phospholipid metabolism in tumour cells. The PDE signal in the liver comes mainly from phospholipid bilayers, but also contains contributions from glycerophosphocholine (GPC) and glycerophosphoethanolamine (GPE), which are breakdown products of phospholipids.

Localized MR spectra were obtained as previously described (Dixon et al, 1992), using a modification of the phase modulated rotating frame imaging technique. The spectra were obtained on a 1 m bore, 2.0 T superconducting magnet (Oxford Magnet Technology, Oxford, UK) interfaced to an Avance spectrometer (Bruker, Coventry, UK) operating at 34 MHz for phosphorus. Each patient was positioned so that the liver was over the coil centre. Metabolite levels were measured with a double surface coil in which the receiver coil (7 cm diameter) was placed forward of, and isolated from the transmitter coil (15 cm diameter). An interpulse delay of 2 s was used, and a pulse width of 90° in the liver. The phosphorus image took 17 min to collect, and the whole study completed in 30–40 min.

After Fourier transformation, the $^{31}P$ MR image consisted of spectra from disc-shaped regions parallel to the surface coil, and at increasing depths into the body. Although the exact volume of tissue that gave rise to the signal in a particular row of the image was not known, the receiver coil was small enough in diameter to ensure that the liver covered the entire sensitive volume of the coil. A row from approximately 1 cm into the liver was selected for quantification. Metabolite signal areas were measured by fitting the signals to Lorentzian and Gaussian lines, after baseline correction. The signal from ATP ($\gamma$-P) was used to measure ATP since the ATP ($\beta$-P) signal was distorted by off-resonance effects, and the ATP ($\alpha$-P) is known to contain contributions from other metabolites.

Patients underwent MRS studies as follows: study 1: pre-infusion (either the day before, or the morning of the first infusion). Study 2: started within 1 h of end of BW12C infusion. Study 3: 1 week later, started within 1 h of end of BW12C+MMC infusion. Study 4: 1–2 weeks later, control study.

### Statistical analysis

To ensure a low probability of rejecting a treatment that is active in 20% of patients, at least 14 patients were treated, according to previously described principles (Gehan, 1961). Comparison of haemoglobin electrophoresis data was performed by paired *t*-tests. Data from the MRS studies were compared by paired *t*-tests and one-way analysis of variance.

### RESULTS

Seventeen patients with hepatic metastases from a colorectal primary entered the study. All patients had previously received chemotherapy with 5-FU, given in a variety of different regimens. Six patients were treated in the adjuvant setting, one of whom progressed on treatment. Eleven patients were treated for metastatic disease with no objective responses, five patients had stable disease (lasting for between 1 and 6 months after their final cycle of 5-FU) and six progressed. In addition, five patients had received radiotherapy, four as adjuvant treatment to the pelvis and one to pelvic disease. The patient characteristics are shown in Table 1.

**Table 1** Patient characteristics

| | |
|---|---|
| Total number treated | 17 |
| Median age in years (range) | 56 (37–67) |
| Male/Female | 10/7 |
| Site of primary | |
| Colon | 10 |
| Rectum | 7 |
| Previous treatment | |
| Surgical resection | 16 |
| 5-FU chemotherapy | 17 (6 adjuvant) |
| Radiotherapy | 5 |
| ECOG performance status | |
| (No. of patients) | 0 (12) |
| | 1 (5) |

**Table 2** Toxicities associated with treatment

| CTC grade | 1 | 2 | 3 | 4 |
|---|---|---|---|---|
| Vein pain | 17 | 0 | 0 | 0 |
| Thromboembolism | 0 | 0 | 0 | 1 |
| Fever | 0 | 2 | 0 | 0 |
| Altered taste | 3 | 0 | 0 | 0 |
| Headache | 4 | 0 | 0 | 0 |
| Fatigue | 7 | 0 | 0 | 0 |
| Nausea/vomiting | 8 | 1 | 0 | 0 |
| Diarrhoea | 1 | 2 | 0 | 0 |
| Mucositis | 1 | 0 | 0 | 0 |
| Musculoskeletal pain | 2 | 0 | 0 | 0 |

Maximal grade toxicities shown.

### Responses

Of 17 patients treated, 15 were evaluable for disease response. Two patients could not be evaluated. One died suddenly 20 days following cycle 1b and one was withdrawn from the study due to toxicity (grade II fever) after cycle 2. This patient continued treatment with MMC alone and the temperature did not recur. There were no responses to treatment and all 15 evaluable patients stopped treatment because of progressive disease. The median number of treatment cycles was 2 (range 1–4).

### Toxicity

The toxicities associated with treatment are shown in Table 2. Vein pain (grade I) occurred in all patients during the loading dose infusion, while the BW12C was administered at high concentration. The pain started around 10 min into the infusion in the cannulated vein, was spasmodic in nature and stopped when the loading dose was finished. No patients experienced vein pain during the maintenance infusion, nor were there any signs of phlebitis. One patient came off study after cycle 1b following a pulmonary embolism diagnosed on ventilation/perfusion scanning which presented as moderate dyspnoea and right sided lower chest pain. Two patients

© 2000 Cancer Research Campaign

**Table 3** Percentage oxyhaemoglobin-BW12C complex with BW12C treatment

| Treatment cycle | 3 h | | | 5 h | | |
|---|---|---|---|---|---|---|
| | Range | Mean | s.d. | Range | Mean | s.d. |
| 1a | 41.0–69.1 | 54.4 | 9.8 | 42.5–60.0 | 53.4 | 6.1 |
| 1b | 53.8–61.9 | 57.9 | 2.6 | 46.1–61.9* | 55.8 | 5.8 |

s.d., Standard deviation. Haemoglobin electrophoresis was performed in eight patients for each time point except * at which there were 7. No complexed haemoglobin was seen at time zero. The table shows the percentage of oxyhaemoglobin-BW12C complex to total haemoglobin at 3 and 5 h after the start of the loading infusion of BW12C in cycles 1a and 1b. There were no significant differences in the percentage of complexed haemoglobin either between time points within a cycle or between cycles at the 3 or 5 h time points.

**Table 4** Subject details for associated MRS study

| | Patients | Controls |
|---|---|---|
| Number of subjects | 8 | 10 |
| Median age in years (range) | 54 (28–67) | 35 (20–72) |
| Male/Female | 6/2 | 5/5 |
| Site of primary | | |
| Colorectal | 5 | |
| Breast | 2 | |
| Lung (squamous) | 1 | |

**Table 5** Mean values of metabolite ratios for the $^{31}$P MRS liver studies

| | Study 1 | s.d. | Study 2 | s.d. | Study 3 | s.d. | Study 4 | s.d. |
|---|---|---|---|---|---|---|---|---|
| PME/ATP | 0.82 | 0.23 | 0.86 | 0.33 | 0.81 | 0.12 | 0.94 | 0.31 |
| Pi/ATP | 0.72 | 0.12 | 0.68 | 0.21 | 0.66 | 0.12 | 0.76 | 0.13 |
| PDE/ATP | 1.79 | 0.35 | 1.76 | 0.36 | 1.92 | 0.26 | 1.82 | 0.38 |
| PME/PDE | 0.46 | 0.11 | 0.51 | 0.24 | 0.42 | 0.04 | 0.51 | 0.07 |
| No. of subjects | 8 | | 8 | | 6 | | 4 | |

| | Controls | s.d. | Significance compared to study 1 (ANOVA) |
|---|---|---|---|
| PME/ATP | 0.61 | 0.15 | 0.036 |
| Pi/ATP | 0.71 | 0.10 | 0.840 |
| PDE/ATP | 1.66 | 0.19 | 0.342 |
| PME/PDE | 0.37 | 0.09 | 0.049 |
| No. of subjects | 10 | | |

PME, phosphomonoester; Pi, inorganic phosphate, PDE, phosphodiester; ATP, adenosine triphosphate. s.d., Standard deviation. Study 1: pre-infusion (either the day before, or the morning of the first infusion); Study 2: started within 1 h of end of BW12C infusion; Study 3: 1 week later, started within 1 h of end of BW12C + MMC infusion; Study 4: 1–2 weeks later, control study. The metabolite ratios provide information about the metabolic activity within the liver; the PME/ATP and PME/PDE ratios are thought to relate to the amount of tumour in the liver, and the Pi/ATP ratio gives an indication of the energetic status of the liver. The PDE/ATP ratio reflects the amount of endoplasmic reticulum within the liver. There were no significant differences in any of the metabolite ratios between any of the patient studies, but the PME/ATP and PME/PDE ratios were significantly higher in the patients than in controls.

developed fevers above 39°C, one during and one shortly following the BW12C infusion in cycle Ib. In both patients the fever settled overnight, however, one of these patients also had rigors and both the pyrexia and rigors recurred following the second cycle. The patient was therefore withdrawn from the study.

Other toxicities included grade 1 headache, grade 1 fatigue and transient nausea/vomiting, as observed in the phase I study. The dose-limiting toxicities from the phase I study (myocardial ischaemia and altered conscious level) was not observed, neither was tachycardia, also seen in the earlier study. One patient experienced mild chest pain after the end of the BW12C infusion (cycle Ia), lasting 15 min. The pain was musculoskeletal rather than cardiac in nature. There were no associated symptoms or signs of cardiac ischaemia and electrocardiograms before and during a subsequent cycle were normal. Another patient described 24 h of backache after cycle 1b. Mucositis (one patient), altered taste and diarrhoea (three patients each), also occurred but were mild (grade ≤ 2).

### Haemoglobin electrophoresis

Haemoglobin electrophoresis studies were performed in eight patients (Table 3). No abnormal haemoglobins were observed in the baseline pre-BW12C treatment samples. A fast moving band was observed in all samples obtained 3 and 5 h after the start of the loading infusion, consistent with the BW12C-oxyhaemoglobin complex (Philip et al, 1993). This accounted for above 50% of the total haemoglobin after 3 and 5 h of treatment (Table 3). There were no significant differences in the percentage of complexed haemoglobin at 3 and 5 h, between cycles 1a and 1b, nor between the 3 and 5 h samples obtained during the same cycle.

### Magnetic resonance spectroscopy

Eight patients were studied, five had hepatic metastases from colorectal primaries, and three with hepatic metastases from other tumour types (Table 4). These three patients were treated as per protocol. Eight patients took part in studies 1 and 2; six went on to

© 2000 Cancer Research Campaign study 3 and four patients completed all four studies. Ten control subjects (Table 4) were also studied by the same MRS protocol (Table 5).

For the patient group, there were no significant changes in any of the measured parameters from one study to the next, either as a group or on an individual basis (that is, there was no trend for the patients' metabolite ratios to increase or decrease between particular studies). Neither was there any change in pH, as calculated from the frequency of the Pi signal, relative to the ATP (α-P) signal. In comparison to the control group, the only significant differences were in the PME/ATP and PME/PDE metabolite ratios, both of which were elevated in patients.

### DISCUSSION

In this phase II study of combined MMC and BW12C treatment, there were no disease responses. Neither was there other evidence that the drug combination modified tumour growth, such as a high incidence of stable disease after treatment. All the patients had previously received 5-FU-based chemotherapy. Responses to MMC as a second line agent after 5-FU are likely to be low. This is suggested by several randomized comparisons of 5-FU to 5-FU plus MMC for treating colorectal cancer which failed to show additive effects (Buroker et al, 1978; Richards et al, 1986), although a small additive effect on response rather than survival was reported (Ross et al, 1997). However, only one response was observed in 72 patients treated with MMC after 5-FU in phase II trials (Hartmann et al, 1998; Migeod et al, 1988; Zaniboni et al, 1995). The current study was therefore a stringent test of the MMC/BW12C combination. Nonetheless, in a phase I trial of BW12C + MMC, 2/26 patients with advanced gastrointestinal cancer attained disease responses (Dennis et al, 1993).

There are at least two reductive pathways by which MMC is activated. The first pathway includes P450 reductase and the second, DT-diaphorase (Mikami et al, 1996). Colon cancer cell lines, mainly from the National Cancer Institute (NCI) human tumour cell panel, generally had high DT-diaphorase levels (Fitzsimmons et al, 1996). The sensitivity of tumour cell lines to MMC correlates with levels of these enzymes, particularly DT-diaphorase (Fitzsimmons et al, 1996; Mikami et al, 1996). However, under hypoxic conditions, DT-diaphorase-rich HT29 cells showed little hypoxic sensitization to mitomycin C (Plumb and Workman, 1994). Furthermore, it is possible that there was insufficient tumour hypoxia to activate MMC, since there are data showing that very marked hypoxia is required to augment MMC cytotoxicity (Brown, 1998). These factors might explain why, despite the possibility of increased tumour hypoxia using BW12C, responses to MMC were not observed.

The aim of this study was to augment MMC activation selectively within the tumour. Haemoglobin electrophoresis showed a fast running BW12C-oxyhaemoglobin band that accounted for a mean of over 50% of the total haemoglobin during the time of BW12C administration. Data from the earlier phase I study indicate that this degree of binding will have been sufficient to cause left shifting of the OSC over 5 half-lives of MMC and therefore maintained for over 95% of the exposure to MMC (Philip et al, 1993). There was no significant difference in the percentage of complexed haemoglobin at 3 and 5 h, between cycles 1a and 1b, indicating that the process is not affected by MMC. The effect of BW12C on tissue oxygen delivery is dependent on the partial pressure of oxygen, and is most marked at low oxygen pressures (Beddell et al, 1984). Hypoxia in tumours varies, and although not defined in colorectal tumours clinically, would be expected to be around 1.5 kPa (Brizel et al, 1997). Based on correlations between BW12C-oxyhaemoglobin levels and oxygen saturation, the degree of BW12C-oxyhaemoglobin complexing observed in this study would be associated with at least a 50% reduction in oxygen delivery to hypoxic tissues, and this percentage reduction in oxygen delivery would rise much more than proportionally as the degree of tumour hypoxia increases (Beddell et al, 1984). This is a function of the sigmoid shape of the OSC. Hence, at least theoretically, hypoxic areas of tumours would have been rendered intensely hypoxic, whereas relatively normoxic tissue would have been much less affected by BW12C.

BW12C induces tissue hypoxia and necrosis, as well as radioresistance. Although these effects are presumed secondary to effects of the drug on the OSC, there is evidence that these effects could in part be mediated by BW12C-induced reductions in tumour blood flow. Falk et al showed that the hypoxic effect of BW12C could be due to a reduction in tumour blood flow rather than hypoxia per se (Falk et al, 1994). They also suggested that the radio-protective effect of BW12C was due to reduced tumour perfusion. Others have also shown that BW12C can reduce tissue perfusion (Honess et al, 1991). If BW12C does reduce tumour blood flow, any bio-reductive effect on MMC would be countered by reduced drug delivery. This could provide another explanation for the absence of tumour effects observed in the current study.

The toxicities in this study were low. All patients developed vein pain, which is related to drug concentration in the loading solution (Philip et al, 1993). Other side-effects were headache, fatigue and nausea/vomiting, which did not exceed CTC grade II toxicity levels. One patient aged 36 died suddenly 20 days after cycle 1b. The cause of death was unknown, but if due to myocardial infarction it seems unlikely that BW12C contributed to this because its effect on the OSC is short (Fitzharris et al, 1985), and in our previous study we showed that the haemoglobin band consistent with the BW12C-oxyhaemoglobin complex had disappeared the day after treatment (Philip et al, 1993). There was no evidence of myocardial ischaemia or alteration in conscious state, which were the dose-limiting toxicities in the phase I study. Nor was there any haematological toxicity, or mucositis. Hence there was no evidence that BW12C potentiates MMC toxicity.

The $^{31}$P MR spectra of the patients' livers showed no significant changes between the studies, either when mean values for the whole patient group were compared, or when the individual changes were compared in a pairwise fashion. We saw no bioenergetic changes, as assessed from the Pi/ATP ratio and the pH, between the initial studies, and the studies immediately following infusion of BW12C with or without MMC. The Pi/ATP ratio was not significantly different from the same ratio in healthy controls. This indicates that BW12C did not significantly alter the energetic status of the liver. This is perhaps surprising, given that the energetics of muscle was altered by this drug (Philip et al, 1993), but may be explained by the fact that the changes in muscle were seen after exercise and not in the resting muscle, and we were effectively looking at the 'resting' liver, in that it had not been stressed by, for instance, administering a metabolic load (e.g. fructose). In addition and as discussed, because of the sigmoid shape of the OSC, BW12C induces most hypoxia in areas that are already hypoxic. The liver has a large blood supply, which may have

© 2000 Cancer Research Campaign increased to compensate for any shortage of oxygen. Also ATP levels can be maintained under hypoxic conditions by a variety of mechanisms (Hochachka et al, 1996)

There were no significant changes in the ratios of PME to any other metabolite following the infusions, which suggests that there was no change of the amount of tumour in the volume of liver sampled. The PME/ATP and PME/PDE ratios in patients were significantly higher than in control subjects, confirming that MRS could detect hepatic metastases. The PME signal is much greater in tumours than in normal liver (Negendank, 1992), and so the signal from PME in the spectra will largely reflect the amount of tumour in the volume sampled. The PME signal in many tumours has been found to be largely composed of phosphoethanolamine, which is a precursor of phospholipids in membranes (Ruiz-Cabello and Cohen, 1992; Dixon, 1996). It is not known why phosphoethanolamine reaches high concentrations (several millimolar) in tumours, but it seems to be generally increased in rapidly dividing tissue, including the regenerating liver. It has been found in a number of studies (Dixon et al, 1991; Negendank, 1992) that following chemotherapy the PME signal of tumours decreases in tumours responding to the therapy. The fact that the PME/ATP and PME/PDE ratios in this study did not alter significantly therefore suggests that the metastatic tumours in the liver did not respond to BW12C and MMC therapy, which is in keeping with the clinical course of the patients.

The current study, and our previous phase I data indicate that the principle of combining a hypoxically activated drug with an agent that stabilizes the OSC to increase tissue hypoxia is clinically feasible, achieves biological effects on tissue oxygen delivery and does not cause undue toxicity. The absence of tumour responses seems likely to be due to the population of patients studied, although there is a possibility that an effect of BW12C in reducing tumour perfusion counters any bioreductive effect. It is also possible that there was insufficient hypoxic activation of MMC. Studies combining BW12C with drugs such as AQ4N and tirapazamine (Patterson, 1993; Workman and Stratford, 1993) which have a much higher hypoxic:normoxic ratio of activity would be of considerable interest. If feasible, measurement of tumour levels of enzymes involved in drug bioreduction would provide additional insight on efficacy.

### REFERENCES

Adams GE, Barnes, DW du Boulay C, Loutit JF, Cole S, Sheldon PW, Stratford IJ, van den Aardweg GJ, Hopewell JW, White RD, Kneen G, Nethersell ABW and Edwards JC (1986) Induction of hypoxia in normal and malignant tissues by changing the oxygen affinity of hemoglobin: implications for therapy. *Int J Radiat Oncol Biol Phys* **12**: 1299–1302

Adams GE, Hasan NM and Joiner MC (1997) The Klaas Breur Lecture. Radiation, hypoxia and genetic stimulation: implications for future therapies. *Radiother Oncol* **44**: 101–109

Beddell CR, Goodford PJ, Kneen G, White RD, Wilkinson S and Wootton R (1984) Substituted benzaldehydes designed to increase the oxygen affinity of human haemoglobin and inhibit the sickling of sickle erythrocytes. *Br J Pharmacol* **82**: 397–407

Brizel DM, Sibley GS, Prosnitz LR, Scher RL and Dewhirst MW (1997) Tumor hypoxia adversely affects the prognosis of carcinoma of the head and neck. *Int J Radiat Oncol Biol Phys* **38**: 285–289

Brown JM (1998) The potential benefit of hypoxic cytotoxins in radio-oncology. In: *Blood Perfusion and Micro-environment in Human Tumours: Implications for Clinical Radio-oncology*, Brady LW and Heilmann H-P (eds), pp. 219–229. Springer-Verlag: Berlin

Buroker T, Kim PN, Groppe C, McCracken J, O'Bryan R, Panettiere F, Coltman C, Bottomley R, Wilson H, Bonnet J, Thigpen T, Vaitkevicius VK, Hoogstraten B and Heilbrun L (1978) 5-FU infusion with mitomycin C versus 5-FU infusion with methyl-CCNU in the treatment of advanced colon cancer: a Southwest Oncology Group Study. *Cancer* **42**: 1228–1233

Cole S and Robbins L (1989) Manipulation of oxygenation in a human tumour xenograft with BW12C or hydralazine: effects on responses to radiation and to the bioreductive cytotoxicity of misonidazole or RSU-1069. *Radiother Oncol* **16**: 235–243

Dennis IF, Ramsay JR, Workman P and Bleehen NM (1993) Pharmacokinetics of BW12C and mitomycin C, given in combination in a phase 1 study in patients with advanced gastrointestinal cancer. *Cancer Chemother Pharmacol* **32**: 67–72

Dixon RM (1996) Phosphatidylethanolamine synthesis in the normal and lymphomatous mouse liver; a 13C NMR study. *Anticancer Res* **16**: 1351–1356

Dixon RM, Angus PW, Rajagopalan B and Radda GK (1991) Abnormal phosphomonoester signals in 31P MR spectra from patients with hepatic lymphoma. A possible marker of liver infiltration and response to chemotherapy. *Br J Cancer* **63**: 953–958

Dixon RM, Angus PW, Rajagopalan B and Radda GK (1992) 31P magnetic resonance spectroscopy detects a functional abnormality in liver metabolism after acetaminophen poisoning. *Hepatology* **16**: 943–948

Falk SJ, Ramsay JR, Ward R, Miles K, Dixon AK and Bleehen NM (1994) BW12C perturbs normal and tumour tissue oxygenation and blood flow in man. *Radiother Oncol* **32**: 210–217

Fitzharris P, McLean AE, Sparks RG, Weatherley BC, White RD and Wooton R. (1985) The effects in volunteers of BW12C, a compound designed to left-shift the blood-oxygen saturation curve. *Br J Clin Pharmacol* **19**: 471–481

Fitzsimmons SA, Workman P, Grever M, Paull K, Camalier R and Lewis AD (1996) Reductase enzyme expression across the National Cancer Institute Tumor cell line panel: correlation with sensitivity to mitomycin C and EO9. *Journal of the National Cancer Institute* **88**: 259–269

Gehan A (1961) The determination of the number of patients required in a preliminary and a follow-up trial of a new chemotherapeutic agent. *J Chronic Dis* **13**: 346–353

Hartmann JT, Harstrick A, Daikeler T, Kollmannsberger C, Muller C, Seeber S, Kanz L and Bokemeyer C (1998) Phase II study of continuous 120 h infusion of mitomycin C as salvage chemotherapy in patients with progressive or rapidly recurrent colorectal cancer. *Anticancer Drugs* **9**: 427–431

Hochachka PW, Buck LT, Doll CJ and Land SC (1996) Unifying theory of hypoxia tolerance: molecular/metabolic defense and rescue mechanisms for surviving oxygen lack. *Proc Natl Acad Sci USA* **93**: 9493–9498.

Honess DJ, Hu, DE and Bleehen, NM (1991) BW12C: effects on tumour hypoxia, tumour thermosensitivity and relative tumour and normal tissue perfusion in C3H mice. *Br J Cancer* **64**: 715–722.

Honess DJ, Nethersell AB and Bleehen NM (1992) In vitro and in vivo studies using BW12C: toxicity, haemoglobin modification and effects on the radiosensitivity of normal marrow and RIF-1 tumours in mice. *Int J Radiat Biol* **61**: 83–94.

Lehmann H and Huntsman RG (1966) *Man's Haemoglobins*. North Holland Publishing Company, Amsterdam.

Migeod F, Gerlach D, Kress M, Hoffmann W, Farroukh R and Seeber S (1988) Sequential treatment of progressive metastatic colorectal cancer with 5-fluorouracil/folinic acid, dipyramidole and mitomycin C. *Onkologie* **2**: 14–20.

Mikami K, Naito M, Tomida A, Yamada M, Sirakusa T and Tsuruo T (1996) DT-diaphorase as a critical determinant of sensitivity to mitomycin C in human colon and gastric carcinoma cell lines. *Cancer Research* **56**: 2823–2826.

Negendank W (1992) Studies of human tumors by MRS: a review. *NMR Biomed* **5**: 303–324.

Patterson LH (1993) Rationale for the use of aliphatic N-oxides of cytotoxic anthraquinones as prodrug DNA binding agents: a new class of bioreductive agent. *Cancer Metastasis Rev* **12**: 119–134.

Philip PA, Thompson CH, Carmichael J, Rea D, Mitchell K, Taylor DJ, Stuart NS, Dennis I, Rajagopalan B, Ganesan T, Radda GK and Harris AL (1993) A phase I study of the left-shifting agent BW12C79 plus mitomycin C and the effect on the skeletal muscle metabolism using 31P magnetic resonance spectroscopy. *Cancer Res* **53**: 5649–5653.

Plumb JA and Workman P (1994) Unusually marked hypoxic sensitization to indoloquinone EO9 and mitomycin C in a human colon-tumour cell line that lacks DT-diaphorase activity. *Int J Cancer* **56**: 134–139.

Richards F, Case LD, White DR, Muss HB, Spurr CL, Jackson DV, Cooper MR, Zekan P, Cruz J, Stuart JJ and et al. (1986) Combination chemotherapy (5-fluorouracil, methyl-CCNU, mitomycin C) versus 5-fluorouracil alone for advanced previously untreated colorectal carcinoma. A phase III study of the Piedmont Oncology Association. *J Clin Oncol* **4**: 565–570.

Ross P, Norman A, Cunningham D, Webb A, Iveson T, Padhani A, Prendiville J, Watson M, Massey A, Popescu R and Oates J (1997) A prospective randomised

*© 2000 Cancer Research Campaign*

trial of protracted venous infusion 5-fluorouracil with or without mitomycin C in advanced colorectal cancer. *Ann Oncol* **8**: 995–1001.
Ruiz-Cabello J and Cohen JS (1992) Phospholipid metabolites as indicators of cancer cell function. *NMR Biomed* **5**: 226–233.
Sartorelli AC, Hodnick WF, Belcourt MF, Tomasz M, Haffty B, Fischer JJ and Rockwell S (1994) Mitomycin C: a prototype bioreductive agent. *Oncol Res* **6**: 501–508.
Workman P and Stratford IJ (1993) The experimental development of bioreductive drugs and their role in cancer therapy. *Cancer Metastasis Rev* **12**: 73–82.
Zaniboni A, Meriggi F, Alghisi A, Mutti S, Distefano L, Rizzi A, Bettini L, Simoncini E, Marpicati P, Montini E and et al. (1995) Mitomycin-C and lonidamine as second-line therapy for colorectal cancer: a phase II study. *Tumori* **81**: 435–437.

 *© 2000 Cancer Research Campaign*
